# The Impact of Advanced Cardiac Life Support Simulation Training on Medical Student Self-reported Outcomes

**DOI:** 10.7759/cureus.7190

**Published:** 2020-03-06

**Authors:** Kaitlin M Bowers, Jacob Smith, Matthew Robinson, Andrew Kalnow, Rich Latham, Andrew Little

**Affiliations:** 1 Emergency Medicine, Hilton Head Hospital, Hilton Head Island, USA; 2 Surgery, Carilion Clinic, Roanoke, USA; 3 Emergency Medicine, Mid-Ohio Emergency Services, Columbus, USA; 4 Emergency Medicine, OhioHealth Doctors Hospital, Columbus, USA; 5 Emergency Medicine, Ohio University Heritage College of Osteopathic Medicine, Athens, USA; 6 Simulation, Ohio University College of Osteopathic Medicine, Columbus, USA

**Keywords:** simulation, simulation trainer, acls, undergraduate medical education

## Abstract

Introduction: Simulation has become a well-recognized and innovative tool in medical education. While there has been tremendous growth of simulation curricula at the level of graduate medical education, there have been few studies looking at simulation as a learning tool for undergraduate medical education. The goal of this study was to determine if high-fidelity simulation training impacts medical student perception of knowledge and confidence regarding comprehension and application of advanced cardiac life support (ACLS) algorithms.

Methods: This is a prospective observational survey study of third and fourth year medical students who participated in an ACLS simulation training during their emergency medicine rotation between January 2018 and October 2018. Cases covered several ACLS topics including unstable bradycardia, supraventricular tachycardia and ventricular tachycardia. After each session, students received a short survey to assess their simulation experience pertaining to knowledge and comfort levels with ACLS topics before and after the simulation experience.

Results: A total of 89 students were included in the study with 86.5% of those being fourth year students. There was a significant increase in both knowledge (pre-training 3.17 vs. 4.11 post-training, p<0.001) and comfort scores (pre-training 2.54 vs. 3.74 post-training, p<0.001) after the ACLS simulation training. Overall, 77.5% of students reported an increase in knowledge and 83.1% reported an increase in confidence after the training session.

Conclusions: The study revealed a statistically significant increase in both perceived knowledge and comfort and confidence of medical students after high-fidelity simulation using ACLS scenarios.

## Introduction

Simulation is becoming a well-recognized and innovative tool in medical education. Similar to an airline pilot practicing in a simulated environment prior to flying a plane, it is becoming common place for training medical personnel to prove and hone their skills and expertise in a simulation environment. Simulation in the field of medical education has been previously defined as “the artificial representation of a situation, environment, or event that provides an experience for the purposes of learning, evaluation, or research” [1]. The available simulation devices range from specialized task trainers to high-fidelity mannequins with the ability to mimic the pathology and responses of real patients. With the ever-increasing breadth of medical knowledge and limited time in training, proponents of simulation have argued that medicine is moving away from the long-standing apprenticeship model and have concluded that simulation may bridge this gap for necessary skills training [2].

In the field of emergency medicine, there has been tremendous growth of simulation curricula in graduate medical education. A survey by Okuda et al. in 2008 noted 85% of participating emergency medicine residency programs employed high-fidelity mannequins in their education curriculum. A mere five years prior to this in 2003, a similar survey concluded that only 29% of programs included high-fidelity simulation in their training [3]. Based on a survey in 2011 of Clerkship Directors in Emergency Medicine, simulation in undergraduate medical education appears to be highly variable. The survey noted that 78% of students are exposed to high-fidelity simulation in their clerkship and that 67% of students noted prior exposure to high-fidelity simulation in their preclinical years [4]. Multiple studies have shown learner preference of this simulation model of instruction over standard methods. Takayesu et al. published a study in 2006 that subjected 95 clinical medical students to a two-hour high-acuity patient simulation. Overall, 94% of students rated the experience as excellent and 91% felt it should be required training [5].

One of the more anxiety provoking and technically difficult skills to master for a student of emergency medicine is the art of resuscitation. Simulation has been employed to help learners achieve this skill in a safe and constructive environment. This tool has the benefit of immersing a learner in the situation and forces decision making in real time with immediate feedback that would be difficult to accomplish through standard methods of instruction. Studies have evaluated the use of simulation with instruction of advanced cardiac life support (ACLS) to determine impact. Conflicting reports exist over the benefit of high-fidelity simulation in undergraduate medical education for ACLS training. Some researchers have found that simulation in general, whether high fidelity or computer based, is of benefit but neither superior to the other [6]. However, a recent study with 19 medical students noted improved outcomes in ACLS training with high-fidelity models as compared to traditional American Heart Association curriculum [7].

Simulation in medical education has recently become an area of interest among researchers as there is an expanding cache of literature to support its use. However, few studies address medical student perception of their knowledge and skills with simulation as a learning tool. Prior studies have demonstrated improved performance and overall satisfaction with the method of instruction. As medical students progress in their education to residency, self-reflection of weaknesses and comfortability with the procedures and skills taught in simulation is a valuable asset. The goal of this research was to explore how simulation with a high-fidelity mannequin in ACLS scenarios impacts medical student perception of their knowledge of ACLS protocols and their comfort with implementation of these protocols in critically ill patients.

## Materials and methods

This is a prospective anonymous survey study of third and fourth year medical students who participated in simulated ACLS cases as part of their emergency medicine clerkship from January through October 2018. Students were excluded from the study if they did not complete at least one full simulated case or if they were a first or second year medical student. Students were permitted to participate multiple times. 

During each simulated session, students were split into teams of five or less. Each team completed a minimum of two ACLS cases. For the cases, a Laerdal SimMan 3G was utilized in a simulation room set up as a resuscitation room. A team leader was designated for each case. Cases were drawn from four preset scenarios that included two unstable bradycardic patients, supraventricular tachycardia and an unstable wide complex tachycardia. One of the bradycardic cases represented an inferior myocardial infarction and the other was a symptomatic bradycardia that required pacing. The students were encouraged to review ACLS protocols prior to their simulated experience. They were also permitted to have pocket cards and phones with them during the cases. 

After each simulated scenario, the students underwent a debriefing session utilizing advocacy-inquiry style discussion, where they were asked to reflect on their skills and review pertinent educational teaching points. Preceptors received a standardized case packet that included reflection questions and teaching points. Preceptors included emergency medicine faculty and senior emergency medicine residents all of whom underwent a training session with one of the study’s investigators. Preceptors also received training on how to operate the simulation mannequins. 

After each session, the students received an anonymous questionnaire. A cover letter was included informing them that participation in the questionnaire was voluntary and anonymous. The questionnaire addressed previous experience, demographics, baseline knowledge of the subject, strengths, weaknesses and overall comfort with the material after the simulation. 

Upon study completion, data were de-identified and entered into a REDCap secure database for analysis. Demographics and prior experience of medical students who participated were described using frequencies and percentages.

Medical student perception of knowledge and confidence regarding comprehension and use of ACLS protocol both before and after completion of the simulation training were summarized. For continuous variables, data were described using means, standard deviations, medians and ranges for the score on a Likert scale of 1 to 5 and compared using paired t-tests. Also, the proportion of respondents with response 4 (agree) or 5 (strongly agree) were compared before and after training using McNemar’s test. The proportion of respondents with improvement on five-point score was also reported to determine the impact of the training. 

The confidence regarding ACLS protocols was reported using frequencies and percentages of agree/strongly agree responses. The dichotomized confidence level among respondents both before and after training was stratified by respondent characteristics and compared using chi-square or Fisher’s exact tests, as appropriate. 

A p-value of less than 0.05 was considered statistically significant.

## Results

A total of 89 medical students were included in the study with 86.5% of them being fourth years. Of these, 55% were male and 45% were female. The average age of participants was 27.5 years, with students ranging from 23 to 40 years of age. Majority (96.6%) of the students were ACLS certified, 91.9% of whom obtained their certification during medical school. Of the 89 students, 83 were participating in the ACLS simulation training for the first time. Approximately 55% did not hold a job in the medical field prior to medical school (Table 1).

**Table 1:** Demographics and Baseline Characteristics ACLS, advanced cardiac life support; EM, emergency medicine; MS, medical student.

Statistic/Category	Result
Gender	
Female	40 (44.9%)
Male	49 (55.1%)
Age	
Mean±SD	27.58±3.18
Range	23.00 to 40.00
Median	27
Years in training	
MS III	12 (13.5%)
MS IV	77 (86.5%)
ACLS certification	
Yes	86 (96.6%)
No	3 (3.4%)
If yes when	
During medical school	79 (91.9%)
Prior to medical school	6 (7.0%)
No response	1 (1.2%)
How many weeks of EM rotations have you completed	
Fewer than 4 weeks	19 (21.3%)
4 to 8 weeks	25 (28.1%)
9 to 12 weeks	26 (29.2%)
Greater than 12 weeks	19 (21.3%)
Prior to medical school did you have any of the following jobs	
N/A	49 (55.1%)
Emergency department technician	1 (1.1%)
Medic	1 (1.1%)
Paramedic/emergency department technician	11 (12.4%)
Emergency department scribe	22 (24.7%)
No response	5 (5.6%)
Have you participated in ACLS simulation training at Doctors Hospital before	
No	83 (93.3%)
Yes	6 (6.7%)
Which ACLS simulation training cases did you participate in today	
Unstable bradycardia I	84 (94.4%)
Unstable bradycardia II	72 (80.9%)
Unstable supraventricular tachycardia	87 (97.8%)
Unstable ventricular tachycardia	76 (85.4%)

There was a statistically significant difference in reported knowledge scores before and after the simulation cases (p<0.001). The mean score increased from 3.17 (before) to 4.11 (after) with a significant average change of 0.94, almost a whole point of improvement (Table 2). Table 3 also shows a statistically significant change from before to after when dichotomizing to "strongly disagree/disagree/neutral" vs. "agree/strongly agree" (p<0.001), as a significant portion of respondents who were disagreeable/neutral before switched to agreeable after (n=43). Overall, 77.5% of participating students reported some improvement in knowledge after the simulated cases. 

**Table 2:** Does High-fidelity Simulation Training Impact Perception of Knowledge or Confidence? *Paired t-test.

Statistic/Category	Knowledge Result	Confidence Result
Before		
N	89	89
Mean±SD	3.17±0.92	2.54±1.01
Range	1.00 to 5.00	1.00 to 5.00
Median	3	3
After		
N	88	89
Mean±SD	4.11±0.72	3.74±0.76
Range	2.00 to 5.00	1.00 to 5.00
Median	4	4
Difference		
N	88	89
Mean±SD	0.94±0.70	1.20±0.80
Range	-1.00 to 3.00	-1.00 to 3.00
Median	1	1
P-value*	<0.001	<0.001

**Table 3:** Do Trainees Agree That the Sessions Improved Knowledge and Confidence?

Before	Knowledge After		Confidence After	
	Strongly disagree/disagree/neutral	Strongly agree/agree	Strongly disagree/disagree/neutral	Strongly agree/agree
Strongly disagree/disagree/neutral	11	43	26	47
Strongly agree/agree	1	33	1	15
McNemar’s test p-value	<0.001		<0.001	

There was also a statistically significant difference in confidence scores before and after the simulated experience (p<0.001). The mean score went from 2.54 (before) to 3.74 (after) with a significant average change of 1.20 points (Table 2). Table 3 also shows a statistically significant change from before to after when dichotomizing to "strongly disagree/disagree/neutral" vs. "agree/strongly agree" (p<0.001), as a significant portion of respondents who were disagreeable/neutral before switched to agreeable after (n=47). Overall, 83.1% of participating students reported some improvement in confidence after the simulation training. 

Students who reported confidence prior to the ACLS training were more likely to have obtained ACLS training prior to medical school (37.5%) compared to those who reported “disagreeable/neutral” confidence (0%) (p<0.001). Participating students who reported higher levels of confidence after the simulated experience were more likely to be fourth year students (93.5%) compared to those who were “disagreeable/neutral” after (70.4%) (p=0.006).

**Figure 1:**
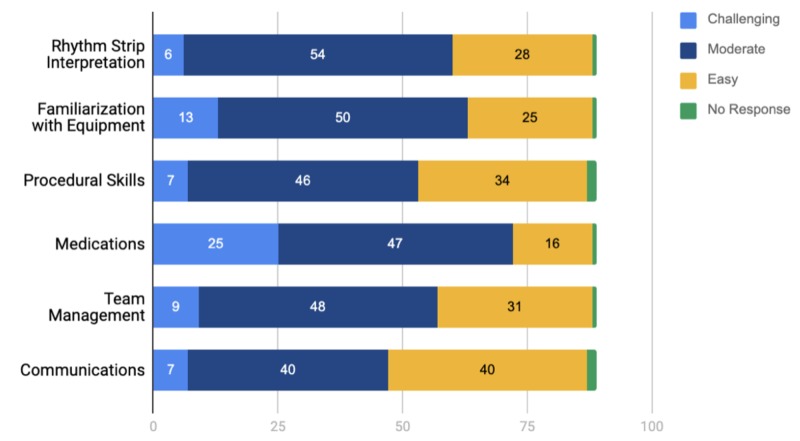
Perceived Level of Learning Difficulty

Figure 1 shows the perceived level of learning difficulty. Medications and familiarization with equipment were the most challenging aspects, compared to communication and procedural skills being reported as the easiest.

## Discussion

This study assesses medical student perceived confidence before and after a dedicated ACLS-simulated training experience. The primary outcome of overall score improvement on learner confidence with ACLS skills suggests that dedicated training during emergency medicine clerkship months can provide an opportunity to better prepare medical students to apply ACLS skills as they advance through training. In our study, we show statistically significant improvement in learner confidence after completing the high-fidelity session. This improvement is noted most drastically in students who had not received formal ACLS training but remains true for students who had already completed a formal course. While we recognize that learner confidence with a skill as complex as ACLS does not translate into performance, it is the foundation for which competency is built. From an educational perspective, early exposure to complex resuscitation in the form of ACLS high-fidelity simulation may provide a significant learning benefit, helping to prepare medical students as they transition to the role of junior physicians. 

Further studies may be strengthened by randomization of the learner to specific scenarios as well as obtaining pre-training and post-training objective data. Adding a control group would also help evaluate if learner perceptions were increased solely due to exposure to the material. Additionally, as this was completed at an institution that hosts an emergency medicine training program, it is possible that auditioning students inflated the effects of the training to appease the residents and attending physicians they may be working shifts with, despite the survey responses being de-identified at blinded to the preceptors. Increasing the number of third year medical student participants would also allow for better data comparison between third and fourth year students, thus potentially supporting earlier simulation incorporation during clinical training years. 

## Conclusions

Our study suggests that high-fidelity simulation is an effective method to increase medical student confidence in ACLS resuscitation of a critically ill patient. While confidence is only one component of delivering quality ACLS care to a patient, this study provides the foundation to further evaluate the impact of such simulated sessions on medical students. Future studies should endeavor to evaluate student performance improvement related to confidence improvement and ultimately look to evaluate patient care impacts of instituting high-fidelity simulation of ACLS early in physician training. 
